# Red Ginseng Extract Ameliorates Autoimmune Arthritis via Regulation of STAT3 Pathway, Th17/Treg Balance, and Osteoclastogenesis in Mice and Human

**DOI:** 10.1155/2014/351856

**Published:** 2014-07-23

**Authors:** JooYeon Jhun, Jennifer Lee, Jae-Kyeong Byun, Eun-Kyung Kim, Jung-Won Woo, Jae-Ho Lee, Seung-Ki Kwok, Ji-Hyeon Ju, Kyung-Su Park, Ho-Youn Kim, Sung Hwan Park, Mi-La Cho

**Affiliations:** ^1^The Rheumatism Research Center, Catholic Research Institute of Medical Science, The Catholic University of Korea, Seoul, Republic of Korea; ^2^Divison of Rheumatology, Department of Internal Medicine, School of Medicine, The Catholic University of Korea, Seoul St. Mary's Hospital, 505 Banpo-dong, Seocho-gu, Seoul 137-701, Republic of Korea; ^3^Conversant Research Consortium in Immunologic Disease, College of Medicine, The Catholic University of Korea, Korea 505 Banpo-Dong, Seocho-Ku, Seoul 137-040, Republic of Korea

## Abstract

Rheumatoid arthritis (RA) is a systemic autoimmune disease characterized by chronic joint inflammation. Red ginseng is a steamed and dried* Panax ginseng* C.A. Meyer, which has been used as alternative medicine for thousands of years. This study was undertaken to investigate the effects of red ginseng extracts (RGE) on autoimmune arthritis in mice and humans and to delineate the underlying mechanism. RGE was orally administered three times a week to mice with arthritis. Oral administration of RGE markedly ameliorated clinical arthritis score and histologically assessed joint inflammation in mice with CIA. A significant reduction in STAT3 phosphorylation and a decrease in the number of Th17 cells were observed with RGE treatment. There was also a marked reduction in RANKL-induced osteoclastogenesis with treatment of RGE. The inhibitory effect of RGE on Th17 differentiation and osteoclastogenesis observed in mice was also confirmed in the subsequent experiments performed using human peripheral blood mononuclear cells. Our findings provide the first evidence that RGE can regulate Th17 and reciprocally promote Treg cells by inhibiting the phosphorylation of STAT3. Therefore, RGE can ameliorate arthritis in mice with CIA by targeting pathogenic Th17 and osteoclast differentiation, suggesting a novel therapy for treatment of RA.

## 1. Introduction

Rheumatoid arthritis (RA) is a systemic autoimmune disease characterized by chronic joint inflammation that can lead to joint destruction and disability. Although the exact molecular mechanism of the RA pathogenesis remains elusive, proinflammatory cytokines including tumor necrosis factor- (TNF-) *α* and interleukin- (IL-) 1*β* and autoreactive T cells are known to play central roles in the development and progression of RA [1]. Although RA was conventionally conceived as Th1-driven inflammatory disease, it is becoming clear that Th17, rather than Th1, is a major pathogenic cell that orchestrates the complex network of the sustained inflammation and disease progression [2–4]. Th17 produces IL-17, which is abundantly expressed in the arthritic joints and neutralization of IL-17 results in attenuation of arthritis [4]. IL-17 not only induces proinflammatory cytokines, but also directly enhances osteoclastogenesis by upregulating receptor activator nuclear kappa ligand (RANKL) on osteoblasts [5], contributing to bone erosion in RA. Recently, Th17 was reported to express RANKL itself as well as directly inducing mature osteoclasts via cell-to-cell contact, substantiating its role in inflammatory bone destruction [6].

The development of Th17 is largely dependent on cytokine milieu. Transforming growth factor- (TGF-) *β* and IL-6 primes initial differentiation and IL-23 promotes functional maturation of Th17 cells [7]. As signal transducer and activator of transcription (STAT) 3 phosphorylation directly regulates retinoic acid receptor-related orphan receptor (ROR) *γ*t, which is the master molecule of Th17, the Janus kinase (JAK)2-STAT3 pathway initiated by IL-6 is essential in the Th17 development. With recent advances in understanding the central role of Th17 in the pathogenesis of RA, novel therapeutics targeting Th17 are being utilized or are still under development. The blockade of IL-6 (Tocilizumab) is currently available on the market, while the JAK2 inhibitor and monoclonal antibody against IL-17 are still under clinical trials among RA patients.

Ginseng, the root of* Panax ginseng* C.A Meyer (Araliaceae) is a perennial herb which has been used as herbal medicine in Eastern Asia for thousands of years [8, 9]. Ginseng has two types of preparation, one is air-dried white ginseng and the other is steamed and sun-dried red ginseng. It contains various active components including ginsenosides, polysaccharides, peptides, polyacetylenic alcohols, and fatty acids [10]. Ginsenosides, in particular, are also called saponin extracts and are known to exert the majority of the pharmacologic effects seen from ginseng. Red ginseng is made from a ginseng plant going through an intensive process of cleaning, steaming, and drying. Heat treatment of ginseng leads to chemical changes of ginsenosides giving them distinct physical characteristics. Major ginsenosides of red ginseng saponin extract were shown to include less polar red ginseng-unique saponins Rg3, Rk1, and Rg5 in a high-performance liquid chromatographic analysis [11]. As supplementation with red ginseng is believed to improve health, numerous studies have been conducted to validate its beneficial effects. They demonstrated that RGEs had anti-inflammatory [12, 13], antidiabetic [14, 15], and anticancer [16] effects. Anti-inflammatory property of RGE leads to the understanding that it may have antiarthritic effect via modulating inflammation. Indeed, accumulating evidence has suggested that ginseng extracts can ameliorate autoimmune arthritis. It was reported that treatment with ginsenoside Rb-1 significantly attenuated arthritis in mice with established CIA [17]. Orally administered RGEs including Rg3, Rk1, and Rg5 as major components also successfully suppressed CIA shown by decreased production of proinflammatory cytokines, matrix metalloproteinase (MMP), and nitrotyrosine in the arthritic joints [11]. More recently, compound K, an active ginsenoside metabolite with a high intestinal absorption rate, was shown to decrease TNF-*α*-induced MMP production in RA-fibroblast-like-synoviocyte (FLS) and suppress osteoclastogenesis by inhibiting the expression of nuclear factor of activated T cells (NFATc1) [18]. However, it has not been suggested whether or not RGE has a regulatory effect on Th17, which is a central pathogenic cell in RA. Given that ginseng was reported to repress STAT3 activation in cancer cells [19], it is plausible to assume that RGE can suppress Th17 by inhibiting STAT3 phosphorylation, enhancing enhanced Treg cells by reciprocal regulation [20].

In the present study, we verified that oral administration of RGE could suppress arthritis in CIA model. To delineate the mechanism underlying the antiarthritic effect in terms of Th17, the effect of RGE on pathogenic Th17 cell differentiation both* in vivo* and* in vitro* was investigated. In addition, the effect of RGE osteoclast formation, which is implicated in bone erosion in RA was examined.

## 2. Methods and Materials

### 2.1. Animals

Six week old male DBA/1J mice were purchased from SLC, Inc., (Shizouka, Japan) and IL-10 knockout (KO) mice in the DBA/1J background were kindly provided by Linda K. Myers (University of Tennessee). Mice were maintained under specific pathogen-free conditions at the institute of Medical Science at the Catholic University of Korea and were provided standard mouse chow (Ralston Purina, St. Louis, MO, USA) and water* ad libitum*. All experimental procedures were examined and approved by the Animal Research Ethics Committee of the Catholic University of Korea (permit number: CUMC:20), which conforms to all National Institutes of Health of the USA guidelines.

### 2.2. Preparation of Red Ginseng Extract

Red ginseng extract (RGE) was kindly provided by the Korea Ginseng Cooperation Daejeon Cooperation (Daejeon, Republic of Korea). RGE yields 4.37% saponins; the main components of ginsenosides were Rb1 (12.6%), Rb2 (6.2%), Rc (6.9%), Rd (3.4%), Re (6.4%), Rf (2.1%), Rg1 (15.8%), and Rg3 (1.4%). The identified constituents are well standardized and qualified by the Korea Ginseng Cooperation. Other constituents in RGE are starch, sugars, fat, fiber, proteins, vitamins, minerals, and so forth.

### 2.3. Induction of CIA and Treatment with Red Ginseng

To induce CIA in mice, 100 *μ*L of an emulsion containing 100 *μ*g bovine type II collagen (CII) and complete Freund's adjuvant (Chondrex, Redmond, WA, USA) was injected intradermally into the base of the tail as the primary immunization. Two weeks later, 100 *μ*g CII, dissolved and emulsified 1 : 1 with incomplete Freund's adjuvant (Difco, Detroit, MI, USA) was administered into the footpad as a booster injection. To assess the influence of RGE on symptom severity in the CIA model, mice were orally treated with 10 mg/kg RGE in saline or with vehicle alone three times a week after booster immunization over the course of 6 weeks. The arthritis index in these mice was scored twice weekly and expressed as the sum of the scores of four limbs.

### 2.4. Measurement of CII-Specific IgG and IgG

Serum levels of CII-specific IgG (Total IgG, IgG1, IgG2a) and IgG (Total IgG, IgG1, IgG2) measurement antibodies were measured using a commercially available ELISA kit (Beathyl Laboratories, Montgomery, TX, USA).

### 2.5. Immunohistochemistry

Mouse joint tissue was fixed in 4% paraformaldehyde, decalcification EDTA bone decalcifier and embedded in paraffin. The section (7 *μ*m) was stained with hematoxylin and eosin, Safranin O, and toluidine blue to detect proteoglycans.

Immunohistochemistry was performed using the Vectastatin ABC kit (Vector Laboratories, Burlingame, CA, USA). Tissue was first incubated with primary antibodies to IL-17, IL-6, IL-1b, TNF-*α*, Nitrotyrosine, NRF2 (Santa Cruz Biotechnology, Santa Cruz, CA, USA), and HO-1, Inos (Abcam, Cambridge Science Park, Cambridge, UK) overnight at 4°C. The sections were counterstained with hematoxylin. Samples were photographed with an Olympus photomicroscope (Tokyo, Japan).

### 2.6. Mouse* In Vitro* Osteoclastogenesis

Isolation of bone-marrow-derived monocyte/macrophage (BMM) cells and differentiation of osteoclast precursor cells (preosteoclasts) was performed as described [21]. Three days later, the nonadherent cells were washed out and preosteoclasts were cultured further in the presence of 10 ng/mL M-CSF, 100 ng/mL RANKL (Peprotech, London, UK), and various concentrations of RGE for four days to generate osteoclasts. On day 2, the medium was replaced with fresh medium containing M-CSF, RANKL, and RGE.

### 2.7. Human in CD4 T Cell Isolation and Differentiation

CD4 T cells were isolated from peripheral blood mononuclear cells (PBMCs) using a CD4 T cell isolation kit (Miltenyi Biotec) according to the manufacturer's instruction. To establish Th17 cell-polarizing conditions, the CD4^+^ T cells were stimulated with plate-bound anti-CD3, anti-CD28, anti-IFN-r, anti-IL-4, IL-1*β* (20 ng/mL), and IL-6 (20 ng/mL) for 3 days. All cytokines were from R&D Systems, with the exception of TGF-*β*.

### 2.8. Intracellular Staining and Flow Cytometry

The following antibodies were used for mouse cells; Th17 cells: PerCP-Cy5.5-conjugated anti-CD4 (eBioscience) and FITC conjugated anti-IL-17A (eBioscience) was used for intracellular staining. The following antibodies were used for human cells: Th17 and Treg cells were from PE-Cy7-conjugated anti-CD4, APC- conjugated anti-CD25 (both from BD Pharmingen), and FITC-conjugated anti-Foxp3, PE-conjugated anti-IL-17 (both from eBioscience).

### 2.9. Enzyme-Linked Immunosorbent Assay (ELISA)

The amount of IL-17, IL-21, IL-22, and IL-10 were measured using a sandwich ELISA (R&D Systems). Absorbance at 405 nm was measured using an ELISA microplate reader (Molecular Devices).

### 2.10. Human* In Vitro* Osteoclastogenesis

The generation of human preosteoclasts was performed as described [21]. After three days, these preosteoclasts were cultured further in the presence of 25 ng/mL M-CSF, 30 ng/mL RANKL, and various concentrations of RGE for nine days to generate osteoclasts. On day 3, the medium was replaced with fresh medium containing M-CSF, RANKL, and RGE. TRAP stain was performed as described [21]. All the subjects gave informed consent before the study. The study received the approval of the institutional review board of Seoul St. Mary's Hospital from all healthy volunteers.

### 2.11. TRAP Staining

A commercial TRAP kit (Sigma, St Louis, ML, USA) was used according to the manufacturer's instructions; however, counterstaining with hematoxylin was omitted. TRAP-positive multinuclear cells (MNCs) containing three or more nuclei were counted as osteoclasts.

### 2.12. Gene Expression Analysis Using Real-Time PCR

PCR amplification and analysis were performed on a Light Cycler 2.0 instrument (Roche Diagnostic, Mannheim, Germany) with software version 4.0. All reactions were performed using LightCycler FastStart DNA master SYBR green I (Takara, Shiga, Japan), according to the manufacturer's instruction. The following primers for mouse samples were used: IL-17, 5′-CCT CAA AGC TCA GCG TGT CC-3′(sense) and 5′-GAG CTC ACT TTT GCG CCA AG-3′(anti-sense); RORrt, 5′-TGT CCT GGG CTA CCC TAC TG-3′(sense) and 5′-GTC CAG GAG TAG GCC ACA TT-3′(antisense); CCR6, 5′-CCA TGA CTG ACG TCT ACC TGT TGA ACA-3′(sense) and 5′-GAA CAG CTC CAG TCC CAT ACC CAG CAG-3′(antisense); Foxp3, 5′-GGC CCTT CTC CAG GAC AGA-3′(sense) and 5′-GCT GAT CAT GGC TGG GTT GT-3′(antisense); SOCS3, 5′-CCT TTG ACA AGC GGA CTC TC-3′(sense) and 5′-GCC AGC ATA AAA ACC CTT CA-3′(antisense); RANK, 5′-TGT ACT TTC GAG CGC AGA TG-3′(sense) and 5′-CCA CAA TGT GTT GCA GTT CC-3′(antisense); MMP9, 5′-CTG TCC AGA CCA AGG GTA CAG CCT-3′(sense) and 5′-GAG GTA TAG TGG GAC ACA TAG TGG-3′(antisense); cathepsin K, 5′-CAG CAG AGG TGT GTA CTA TG-3′(sense) and 5′-GCG TTG TTC TTA CGA GC-3′(antisense); TRAP, 5′-TCC TGG CTC AAA AAG CAG TT-3′(sense) and 5′-ACA TAG CCC ACA CCG TTC TC-3′(antisense). The following primers for human samples were used: RANK, 5′-GCT CTA ACA AAT GTG AAC CAG GA-3′(sense) and 5′-GCC TTG CCT GTA TCA CAA ACT-3′(antisense); MMP9, 5′-CGC AGA CAT CGT CAT CCA GT-3′(sense) and 5′-GGA TTG GCC TTG GAA GAT GA-3′(antisense); cathepsin K, 5′-TGA GGC TTC TCT TGG TGT CCA TAC-3′(sense) and 5′-AAA GGG TGT CAT TAC TGC GGG-3′(antisense); CTR, 5′-TGG TGC CAA CCA CTA TCC CTG A-3′(sense) and 5′-CAC AAG TGC CGC CAT GAC AG-3′(antisense). The level of mRNA expression was normalized to that of *β*-actin.

### 2.13. Immunoblot Analysis

PBMC were cultured with anti-CD3 and anti-CD28 in the presence or absence of RDE for 72 h. Mice splenocytes were cultured with the Th17 condition in the presence or absence of RDE for 72 h. Both cells were then harvested and lysed with lysis buffer. Protein concentration was measured using the Bradford method (Bio-Rad, Herculed, CA, USA). Protein samples were separated using 12% SDS-PAGE and transferred onto nitrocellulose membranes (AmersharmPharmacia Biotech, Piscataway, NJ, USA). For Western blot hybridization, the membrane was preincubated with blocking buffer for 2 h and then incubated with primary antibodies against Total IkBa, p-IkBa, Total ERK, p-ERK, Total STAT5, pSTAT5, Total STAT3, pSTAT3(727), pSTAT3(705) (all from Cell Signaling, Danvers, ma), and *β*-actin for 1 h. After washing, horseradish peroxidase-conjugated secondary antibodies were added, and the membranes were incubated for 1 h at room temperature. After washing, the hybridized bands were detected using an ECL detection kit (Pierce, Rockford, IL, USA) and Hyperfilm (Agfa, Belgium).

### 2.14. Statistical Analysis

Experimental values are presented as mean ± SD of triplicate cultures and representative of experiments performed on three occasions. Statistical significance was determined by Mann-Whitney *U* test or ANOVA with Bonferroni's post-hoc test using the Graphpad Prism (v.5.01). Values of *P* < 0.05 were considered statistically significant, *P < 0.05; **P < 0.01; ***P < 0.001.

## 3. Results

### 3.1. Red Ginseng Extract Suppresses Collagen-Induced Arthritis

**Figure 1:**
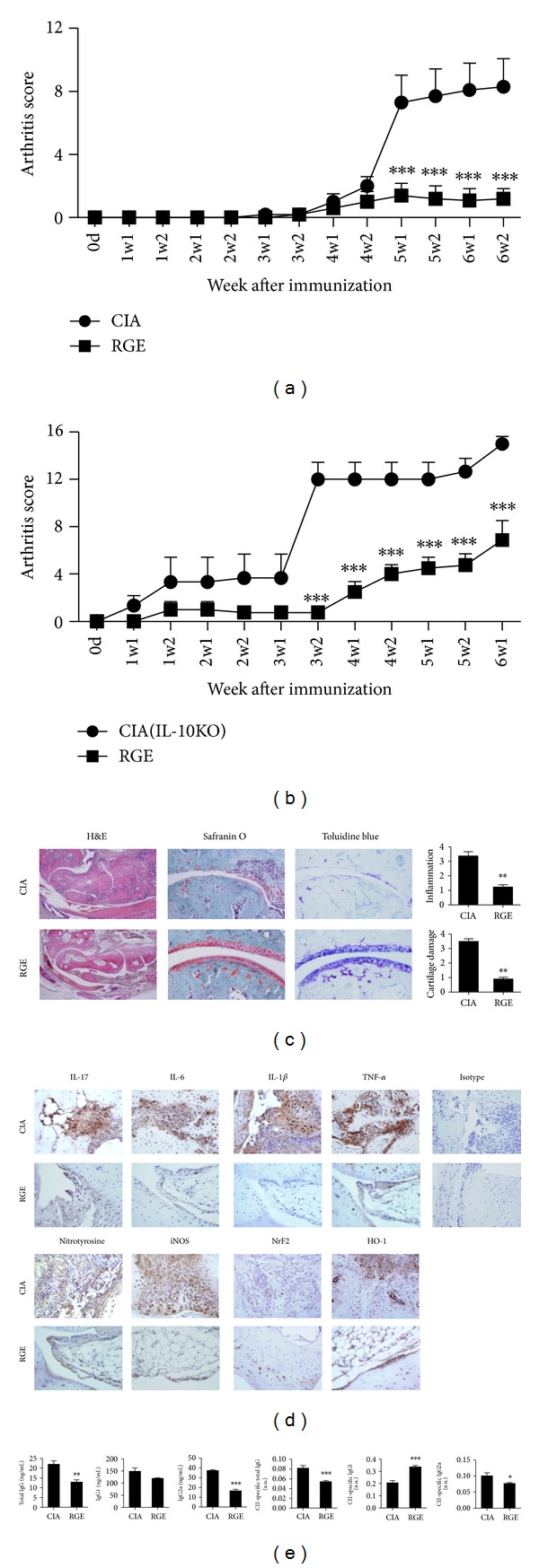
Red ginseng extract suppresses collagen-induced arthritis. Collagen-induced arthritis (CIA) was induced. Mice were orally treated with 10 mg/mL of red ginseng extract (RGE) or vehicle only. (a) Clinical scores and incidence of arthritis in CIA-induced DBA/1J mice. (b) Clinical scores and incidence of arthritis in IL-10 KO mice. (c) Representative histological features of the joint of RGE or vehicle-treated mice with CIA. H&E, safranin O, and toluidine blue staining are shown. (d) Representative immunohistochemical staining of the joint of RGE or vehicle-treated mice with CIA (e) Concentrations of CII-specific IgG and IgG2a in sera of RGE or vehicle-treated mice with CIA were determined by ELISA. Data are presented as the mean ± SD of three independent experiments *P < 0.05, **P < 0.01, and ***P < 0.001 compared to control mice.

Results showed that administration of oral RGE three times a week (10 mg/kg) reduced the arthritic score and arthritis incidence almost completely compared to the oral administration of the vehicle (Figure 1(a)). When CIA was induced among the IL-10 KO mice, there were higher rates of clinical signs and more severe knee and paw injury when compared to the wild type mice. As shown in Figure 1(b), RGE successfully suppressed the arthritic score and arthritis incidence in the CIA mice of the IL-10 knockout background. Histological examination of the joints demonstrated that the paws and ankles of the red ginseng extract-treated mice had a lower degree of inflammation and cartilage damage compared with those of the vehicle-treated mice, as determined on day 49 after immunization (Figure 1(c)). In addition, the red ginseng extract-treated mice expressed markedly lower levels of not only proinflammatory cytokines such as TNF-*α*, IL-1b, IL-6, and IL-17, but also oxidative stress markers such as nitrotyrosine and iNOS as demonstrated by immunohistochemical analysis (Figure 1(d)). The results implicated that antioxidant activity of RGE might contribute to attenuating oxidative stresses in CIA mice. We next examined whether treatment with red ginseng extract would modulate humoral immune responses by assessing Ab production in CIA mice.  Figure 1(e) illustrates that treatment with RGE efficiently attenuated the production of total IgG and IgG2a, the Th1-type Ab, in the sera of CIA mice. The effect of red ginseng extract on the Ag-specific humoral immune responses* in vivo* was also assessed. The serum levels of the CII-specific IgG2a and total IgG were significantly lower in the mice treated with red ginseng extract (Figure 1(e)). Interestingly, the CII-specific IgG1a, the Th2-type Ab, was considerably increased among the red ginseng extract-treated CIA mice (Figure 1(e)).

### 3.2. Red Ginseng Extract Reciprocally Modulates Populations of Regulatory T Cells and Th17 Cells in CIA Mice

**Figure 2:**
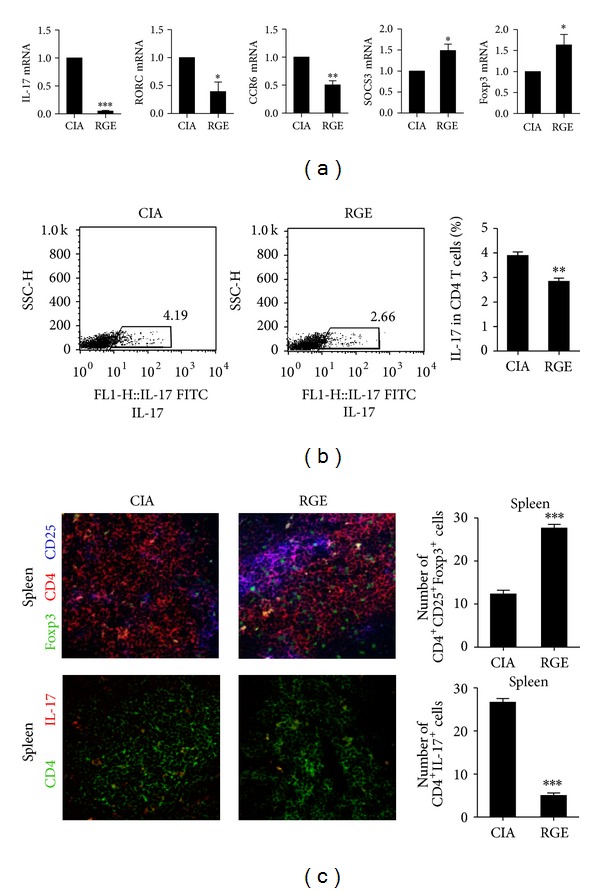
Red ginseng extract reciprocally modulates populations of regulatory T cells and Th17 cells in CIA mice. (a) Messenger RNA was isolated from splenocytes of either red ginseng extract (RGE) or vehicle-treated CIA mice. The mRNA expressions of Th17 cell-related molecules were measured by RT-PCR. (b) The proportion of IL-17-producing CD4^+^ T cells in the splenocytes was analyzed by flow cytometry. (c) Spleens were subjected to immunostaining for CD4^+^CD25^+^Foxp3^+^ or CD4^+^IL-17^+^ cells. The number of cells was counted in four independent quadrants. Data are presented as the mean ± SD of three independent experiments *P < 0.05, **P < 0.01, and ***P < 0.001 compared to control mice.

mRNA from splenocytes of either red ginseng extract-treated CIA mice or vehicle-treated CIA mice was isolated and the expression of Th17 cell- and Treg cell-related markers by RT-PCR was then analyzed. The results showed that the mRNA levels of Th17 cell-related molecules such as IL-17, RORC, and CCR6 were downregulated whereas the mRNA levels of Treg cell-related molecules such as Foxp3 and SOCS3 were upregulated in the red ginseng-treated CIA mice (Figure 2(a)). Furthermore, the red ginseng treatment reduced the number of IL-17-producing CD4^+^ T cells in the splenocytes of CIA mice as analyzed by flow cytometry (Figure 2(b)). Additionally, we measured the numbers of CD4^+^CD25^+^Foxp3^+^ regulatory T cells and CD4^+^IL-17^+^ T cells (Th17 cells) in tissues of spleens and drain lymph nodes by immunofluorescence confocal microscopy. The spleen tissues from the mice treated with red ginseng extract showed an increased number of Foxp3^+^ regulatory T cells and a decreased number of Th17 cells compared with those of the vehicle-treated mice (Figure 2(c)). The number of Th17 cells was also significantly decreased in the tissues of the drain lymph nodes (data not shown), although the number of Treg cells remained unchanged (Figure 2(c)).

### 3.3. Red Ginseng Extract Reduces STAT3 Phosphorylation in the CD4^+^ T Cells in Mice

**Figure 3:**
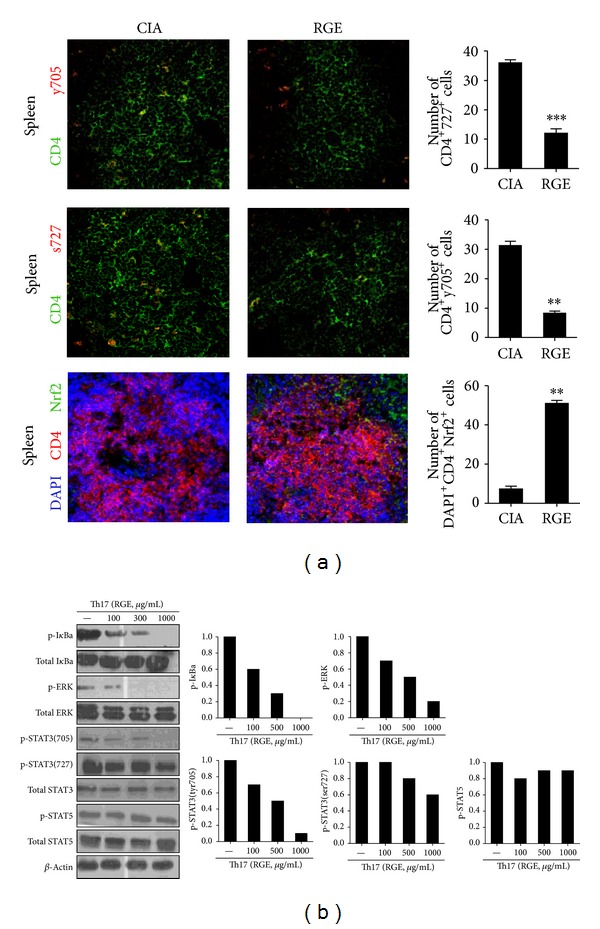
Red ginseng extract reduces STAT3 phosphorylation in the CD4^+^ T cells in mice. (a) Spleens of red ginseng extract or vehicle-treated CIA mice were subjected to immunostaining for CD4^+^pSTAT3y705^+^ or CD4^+^pSTAT3s727^+^ cells. The number of cells was counted in four independent quadrants. (b) Isolated CD4^+^ T cells were cultured in a Th17-polarizing condition for 3 days in the absence or presence of various concentrations of red ginseng extract (RGE). The expression of various signaling molecules was determined by western blotting. Data are presented as the mean ± SD of three independent experiments *P < 0.05, **P < 0.01, and ***P < 0.001 compared to control mice.

The number of pSTAT3-expressing CD4^+^ T cells was indeed decreased in the red ginseng-treated CIA mice compared with those of the vehicle-treated CIA mice (Figure 3(a)). To evaluate whether RGE promotes phosphorylation of STAT3* in vitro*, we performed immunoblot analysis of protein extracts prepared from CD4^+^ T cells isolated from spleens cultured in a Th17-polarizing condition with various concentrations of RGE. RGE dose-dependently decreased the amount of phosphorylated STAT3 at tyrosine 705 under the Th17-generating condition while the total amount of STAT3 remained the same (Figure 3(b)). Phosphorylation of IkB and ERK was also reduced with RGE treatment (Figure 3(b)). After stimulation under conditions favoring the development of Th17 cells, we found that phosphorylation of STAT3 at 705 and 727 was significantly decreased by the addition of red ginseng extract, although phosphorylation of STAT3 at 727 was reduced in a less degree (Figure 3(b)).

### 3.4. Red Ginseng Extract Inhibits Osteoclastogenesis in CIA Mice

**Figure 4:**
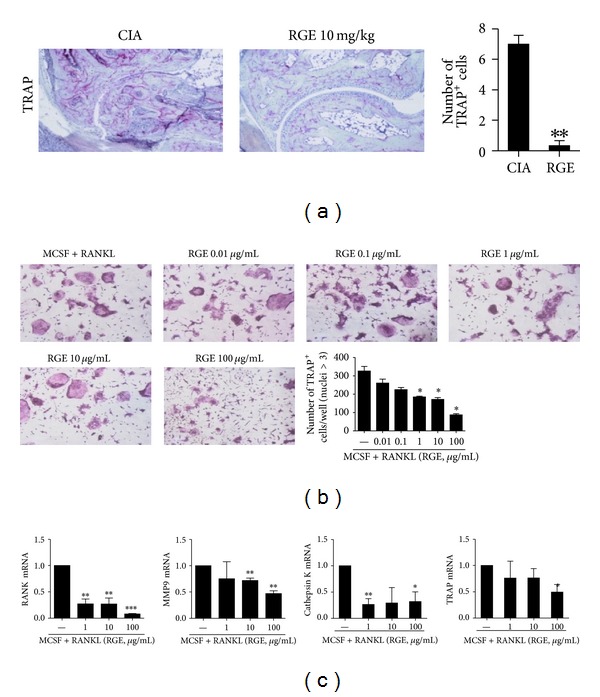
Red ginseng extract inhibits osteoclastogenesis in CIA mice. (a) The number of TRAP^+^ cells in the arthritic joint of red ginseng extract (RGE) or CIA was counted using light microscopy. The BMM cells from the DBA/1J mice were cultured with M-CSF (10 ng/mL) and RANKL (50 ng/mL) in the presence or absence of various concentrations of RGE. (b) Cells were fixed and stained for TRAP (original magnification, ×100). The number of TRAP^+^ cells was counted using light microscopy. The representative photographs from each group are shown. (b) The mRNA expressions of various osteoclastogenic markers such as RANK, MMP9. Cathepsin K and TRAP were analyzed using real time PCR. BMM: bone-marrow-derived monocyte/macrophage; MMP9: matrix metalloproteinase 9. Data are presented as the mean ± SD of three independent experiments *P < 0.05, **P < 0.01, and ***P < 0.001 compared to control mice.

The number of TRAP positive cells was markedly reduced in the joint tissues of RGE-treated mice compared with those of vehicle-treated mice (Figure 4(a)). We next investigated whether RGE would directly inhibit osteoclast formation* in vitro*. The BMM cells were prepared from WT mice and stimulated with M-CSF and/or RANKL to induce osteoclast differentiation. The addition of various concentrations of RGE during the induction of osteoclastogenesis significantly inhibited osteoclast formation in a dose-dependent manner (Figure 4(b)). Transcripts of various osteoclastogenic markers such as RANK, MMP9, cathepsin K, and TRAP were also considerably decreased by the addition of RGE (Figure 4(c)).

### 3.5. Red Ginseng Extract Increased Foxp3-Expressing Regulatory T Cells and Decreased IL-17-Expressing Th17 Cells in Human PBMCs

**Figure 5:**
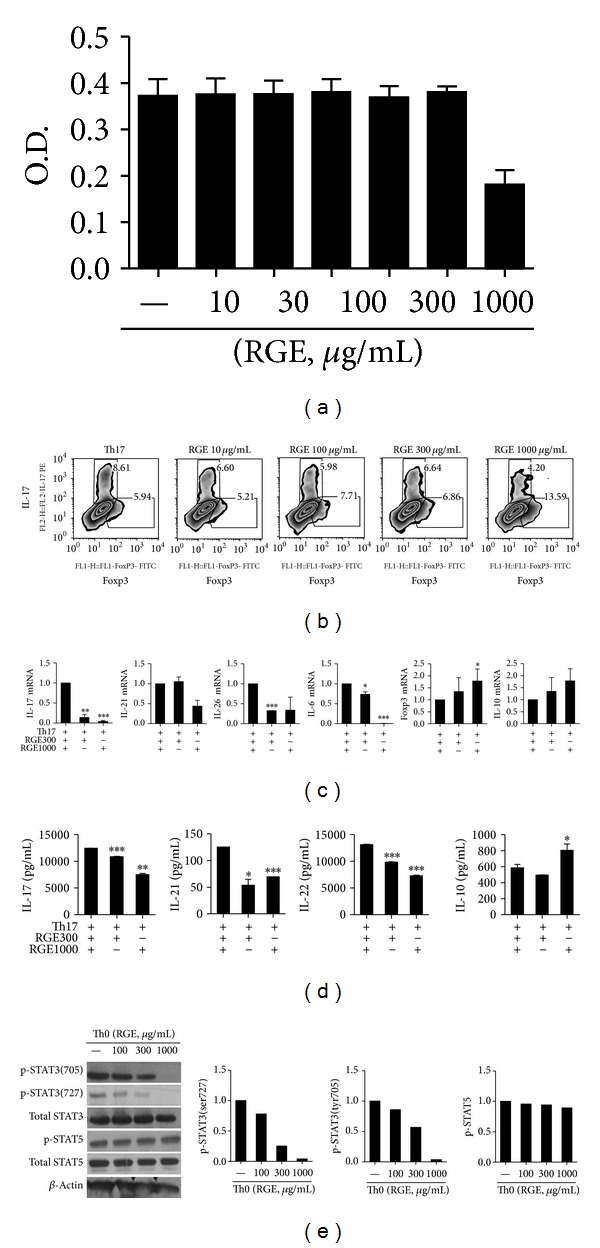
Red ginseng extract increased Foxp3-expressing regulatory T cells and decreased IL-17-expressing Th17 cells in human PBMCs. CD4^+^ T cells were isolated from human peripheral blood and cultured with various concentrations of red ginseng extract (RGE) (a) MTT assay (b) The proportion of IL-17 positive cells and Foxp3 positive cells was analyzed by flow cytometry. (c) The mRNA expressions of Th17 - and Treg-associated molecules were measured by RT-PCR. (d) The concentrations of IL-17, IL-21, and IL-22 in the supernatant were measured by ELISA. (e) The level of total and phosphorylated STAT3 (at Y705 and S727, resp.) and STAT5 was assessed by western blotting. Data are presented as the mean ± SD of three independent experiments *P < 0.05, **P < 0.01, and ***P < 0.001 compared to control.

Concentrations of RGE used in these* in vitro* experiments did not affect cell viability as demonstrated by the MTT assay (Figure 5(a)). Transcripts of Treg-related molecules such as Foxp3, Socs3, and IL-10 significantly increased whereas mRNAs of IL-17 and IL-6 were markedly reduced by red ginseng extract in CD4^+^ T cells of human PBMCs (Figure 5(c)). Additionally, Th17-associated cytokines like IL-26 and IL-21 were also decreased although IL-22 did not change significantly (Figure 5(c)) at the mRNA level. Subsequent flow cytometry analysis confirmed that the populations of Treg and Th17 cells were indeed reciprocally regulated, in which Treg cells were increased and Th17 cells were decreased by RGE in a dose-dependent manner (Figure 5(b)).

CD4^+^ T cells either when activated with anti-CD3/28 antibodies alone (data not shown) or when cultured in a Th17-polarizing condition produced considerably lower levels of IL-17, IL-21, and IL-22 after the treatment with RGE. Interestingly, Treg-associated cytokines, IL-10 was significantly increased by the addition of RGE (Figure 5(d)). Phosphorylation of STAT3 at tyrosine 705 and 727 was markedly reduced with the treatment of 1000 *μ*g/mL red-ginseng extract (Figure 5(e)). However, the amount of pSTAT5 was not affected by RGE treatment.

### 3.6. Red Ginseng Extract Inhibits Osteoclastogenesis in Humans

**Figure 6:**
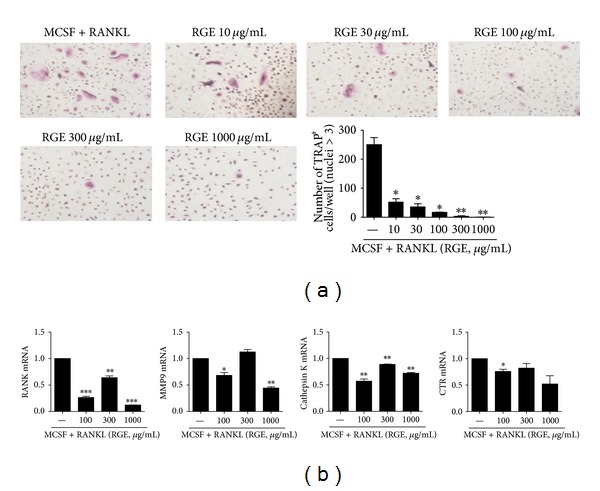
Red ginseng extract inhibits osteoclastogenesis in humans. Human peripheral blood mononuclear cells were cultured with M-CSF (10 ng/mL) and RANKL (50 ng/mL) in the presence or absence of various concentrations of RGE. (a) Cells were fixed and stained for TRAP (original magnification, ×100). The number of TRAP^+^ cells was counted using light microscopy. The representative photographs from each group are shown. (b) The mRNA expressions of various osteoclastogenic markers such as RANK, MMP9. Cathepsin K and CTR were analyzed using real time PCR. BMM: bone-marrow-derived monocyte/macrophage; CTR: calcitonin receptor; MMP9: matrix metalloproteinase 9. Data are presented as the mean ± SD of three independent experiments *P < 0.05, **P < 0.01, and ***P < 0.001 compared to control.

Figure 6(a) displays that RGE treatment effectively prevented human monocytes from differentiating into mature osteoclasts, which was determined by TRAP staining. It also reduced the expressions of osteoclastogenic markers such as the calcitonin receptor (CTR), cathepsin K, and MMP-9 (Figure 6(b)).

## 4. Discussion

In this study, oral administration of RGE ameliorated the clinical arthritis score and the histological severity of joint inflammation in mice with CIA. RGE inhibited differentiation of Th17, which is a main pathogenic T cell in RA, by suppressing phosphorylation of STAT3 and reciprocally increased Treg population. Furthermore, treatment with RGE substantially suppressed osteoclastogenesis, which might contribute to less bone erosion in CIA mice treated with RGE.

A significant reduction in proinflammatory cytokines including IL-17, IL-6, TNF-*α*, and IL-1*β* was observed in the joints of RGE-treated CIA mice. As* in vitro* studies have demonstrated that IL-17 is a powerful inducer of IL-6, TNF-*α*, and IL-1*β* [3], a decrease in Th17 which in turn reduced IL-17 in the arthritic joints seemed to cause the decrease in production of those cytokines. Moreover, decreased production of IL-6 and IL-1b might have contribution to suppressed Th17-IL-17 activation.

As oxidative stress is known to play an important role in RA pathogenesis [22–24], oxidative stress represented by iNOS and nitrotyrosine expression was also measured, which diminished with RGE treatment in CIA. Nrf2 is a transcription factor, activating upon the exposure to ROS. Expression of Nrf2 and HO-1 suppressed regulation of oxidative stress [25]. Activated Nrf2 binds to antioxidant response element (ARE) located in the regulatory regions of the genes coding for antioxidative enzymes such as HO and enhances transcription of the antioxidative enzymes [26]. Thus, decreased expression of Nrf2 and HO in the joints of RGE-treated mice indicates that the oxidative stress was lower than the vehicle-treated mice. Our data corroborate the previously reported antioxidative effect of RGE [27, 28]. In addition to inhibitory effect on Th17, the antioxidative property of RGE seems to exert an additional effect in suppressing CIA.

The main focus of this study was to investigate whether the antiarthritic effect of RGE was mediated by the regulation of pathogenic Th17 cells. As expected, the number of Th17 cells was significantly reduced among the RGE-treated mice with CIA, indicating that RGE suppressed Th17 differentiation in arthritic condition. As mentioned, Development of Th17 cell was caused by activation of STAT3 signal pathway [7]. Therefore, we investigated whether RGE could suppress STAT3 phosphorylation, and discovered that phosphorylation of STAT3 was decreased in RGE-treated mice as demonstrated by immunofluorescence confocal microscopy and in RGE-treated CD4^+^ T cells cultured in Th17 polarizing conditions measured by immunoblotting. These data suggest that RGE might block the signal transduction pathway initiated by binding of IL-6 to its receptor, which activates downstream kinases and subsequently allows the phosphorylation of STAT3. This was consistent with previous report that American ginseng, albeit different genus, dramatically suppressed JAK2-STAT3 activation in aortic smooth muscle cell in rats [29]. Diminished expression of IL-6 also seems to have contributed to the Th17 regulation. To elucidate whether RGE directly regulates IL-6 expression requires future research.

The effect of RGE on Treg as well as Th17 in pathologic inflammatory condition in mice with CIA or in CD4^+^ T cells cultured in Th17 polarizing condition was investigated. Our data showed that the number of Treg cells increased with RGE treatment while Th17 differentiation was suppressed. This was in line with the previous finding that ginsenoside Rp-1 activated Treg cells [30]. However, this simultaneous regulation on Th17 and Treg by red ginseng has never been reported. The notion that Treg is reciprocally regulated with Th17 originates from the report that advocated the plasticity of T cell subsets [31]. Th17 and Treg are thought to have common precursors cell before they are destined to certain effecter cells. As transcription of IL-17 is regulated by competitive binding of pSTAT3 and pSTAT5 [20], and pSTAT5 is a critical transcriptional factor for Foxp3, the master molecule of Treg, the ratio of pSTAT3 and pSTAT5 is expected to be one of determinants for final effector cell type. While RGE significantly reduced pSTAT3, the level of STAT5 phosphorylation was not increased with RGE treatment. Although the RGE failed to increase the amount of pSTAT5, the decreased ratio of pSTAT3/pSTAT5 seemed to contribute to the Th17 suppression and the reciprocal increase in Treg. Namely, the relative reduction in pSTAT3 may have resulted in pSTAT5 binding on promoter site of IL-17 and suppressed IL-17 expression. With the suppression of Th17 differentiation, a shift toward Treg differentiation seemed to be augmented. Although the differentiation into a specific T cell subset is mainly determined by master transcriptional regulator (e.g., ROR*γ*t, Foxp3) and phosphorylation of STATs, recent data indicate that other signaling pathways are also involved in this fine-tuning process. ERK signaling was suggested to be one of those pathways [32] where an inhibition of ERK decreases IL-6-induced ROR*γ*t expression and reciprocally enhances TGF-*β*-induced Foxp3 expression, resulting in decreased Th17/Treg ratio. Hence, the ability of RGE to suppress phosphorylation of ERK as well as STAT3 may be another efficient mechanism by which RGE regulated Th17/Treg balance.

The suppression of osteoclastogenesis by RGE was demonstrated by both* in vivo* and* in vitro*. In CIA joints, the decrease in number of Th17 cells, seems to have resulted in a reduction in TRAP(+) osteoclasts. The inhibitory effect of RGE on osteoclastogenesis* in vitro* was previously reported by He et al. [33]. They demonstrated that ginsenoside Rh2, a component of red ginseng, suppressed osteoclast differentiation by inhibiting RANKL-induced c-fos and NFATc1 expression. Ginsenoside Rh2 also inhibited phosphorylation of ERK and I*κ*B*α*, thereby suppressing NF-*κ*B, which was consistent with our findings observed in CD4^+^ T cells. In addition, these results showed that RGE directly suppressed RANK expression during osteoclastogenesis* in vitro*, hampering RANKL-induced signaling.

The limitation of this study is that the exact chemical composition of RGE used was not analyzed. Therefore, it is unclear which component of RGE is involved in specific molecular event. For instance, one ginsenoside may inhibit STAT3 phosphorylation in Th17 polarizing condition and another ginsenoside may suppress RANK expression during osteoclastogenesis or both effects can be mediated by one ginsenoside. Namely, the antiarthritic effect observed in the present study may be a constellate effect of several ginsenosides. However, red ginseng is generally ingested as a whole rather than any extracted form of specific ingredient. Hence, our data suggest red ginseng, as a whole, can be utilized as a novel therapeutic for RA.

The present study provides the first evidence that RGE can regulate Th17 differentiation. By inhibiting STAT3 phosphorylation, RGE concurrently suppresses Th17 and enhances Treg. This novel mechanism will provide the solid basis that red ginseng, conceiving as an alternative medicine, can be a promising therapeutic with scientific evidence. Furthermore, it can be expected that RGE may have therapeutic potential in other diseases where Th17 plays major role in pathogenesis such as inflammatory bowel disease or graft-versus-host disease. Future research on this topic will be promising.

## 5. Conclusion

In conclusion, our data demonstrate that RGE has a profound inhibitory effect on CIA development as well as its severity. The inhibitory effect appeared to be mediated through reciprocal regulation of Th17 and Treg by suppressive effect on STAT3 phosphorylation of RGE. RGE also directly inhibited osteoclastogenesis which is critical for bone erosion in RA. The regulatory effect was consistently found in experiments using human PBMCs, suggesting that antiarthritic effect of RGE could be applicable to human RA. In addition, RGE has been used widely in alternative medicine with relatively good safety profile. Therefore, RGE may be a novel therapeutic agent for the treatment of RA.
